# Possible Differentiation Between Recurrent Lymph Nodes of Bladder Cancer and Malignant Lymphoma on Ultrasound: A Case Report

**DOI:** 10.7759/cureus.98260

**Published:** 2025-12-01

**Authors:** Hiroki Yamamoto, Shoji Oura

**Affiliations:** 1 Department of Surgery, Kishiwada Tokushukai Hospital, Kishiwada, JPN

**Keywords:** bladder cancer, internal high echoes, malignant lymphoma, scc, sil-2r

## Abstract

In clinical practice, diagnostic physicians often have difficulty distinguishing between malignant lymphomas and recurrent lymph nodes of prior solid malignancies. An 86-year-old man, with a past history of bladder cancer, complained of pain around the left shoulder. Ultrasound showed multiple round masses extending from the left neck to the left axilla, with mixed high and low internal echoes and enhanced posterior echoes. Positron emission tomography showed avid fluorodeoxyglucose uptake, i.e., a maximal standard uptake value (SUV max) of 19. Magnetic resonance imaging of the (peri)axillary nodes showed low signals on T1-weighted images and predominantly very weak high signals on fat-suppressed T2-weighted images. Blood tests showed an elevated squamous cell carcinoma (SCC) antigen level of 9.5 ng/mL and a mildly elevated soluble interleukin-2 receptor (sIL-2R) level of 851 U/mL. Ultrasound findings highly suggested the recurrence of bladder cancer but could not rule out possible malignant lymphoma due to the elevated sIL-2R level.

We, therefore, performed an excisional biopsy of the enlarged but easily resectable lymph node in the axilla. Frozen section of the target lesion showed no lymphoma cells but atypical cells proliferating in a solid fashion, with keratinization, intercellular bridges, and hypo-cellular areas. Immunostaining showed GATA3, p40, and CK5/6 positivities, leading to the diagnosis of SCC metastasis to the lymph node. Due to both the patient’s old age and preference, he did not receive chemotherapy and only underwent radiotherapy to the supra-clavicular, axillary, and intra-abdominal foci for disease control, unfortunately resulting in the discontinuation of radiotherapy shortly thereafter due to severe side effects, including grade 2 diarrhea. The patient, therefore, has been receiving best supportive care for three months after the excisional biopsy. Diagnostic physicians should note that malignant lymphomas rarely have internal high echoes.

## Introduction

Smoking and occupational exposure to carcinogens, e.g., aromatic amines, are well-known risk factors for bladder cancer [1-3]. Bladder cancer, therefore, is three times more common in men than in women and is frequently observed in elderly patients. It is also well known that elderly male smokers are more likely to develop various malignancies, such as lung cancer and esophageal cancer, in addition to bladder cancer.

Malignant lymphoma accounts for more than 50% of hematological malignancies [4,5]. Although malignant lymphoma may arise even in organs without lymph nodes (e.g., brain) [6], it typically develops in lymph node-rich areas, such as the neck, axillae, and inguinal regions. In addition, some patients develop chemotherapy-induced malignant lymphomas (e.g., methotrexate-associated malignant lymphomas) [7]. It is also not uncommon for cancer patients, regardless of whether they are smokers or not, to develop malignant lymphomas in addition to their prior malignancies. Oncologists, therefore, often struggle to make an accurate diagnosis of newly developed masses in presumed lymph nodes when the patients have received curative therapy for prior malignancies.

We herein report a bladder cancer patient with presumed malignant lymph nodes around the left axilla, which were successfully judged as cancer metastasis through pathological component-based image evaluation [8,9].

## Case presentation

**Figure 1:**
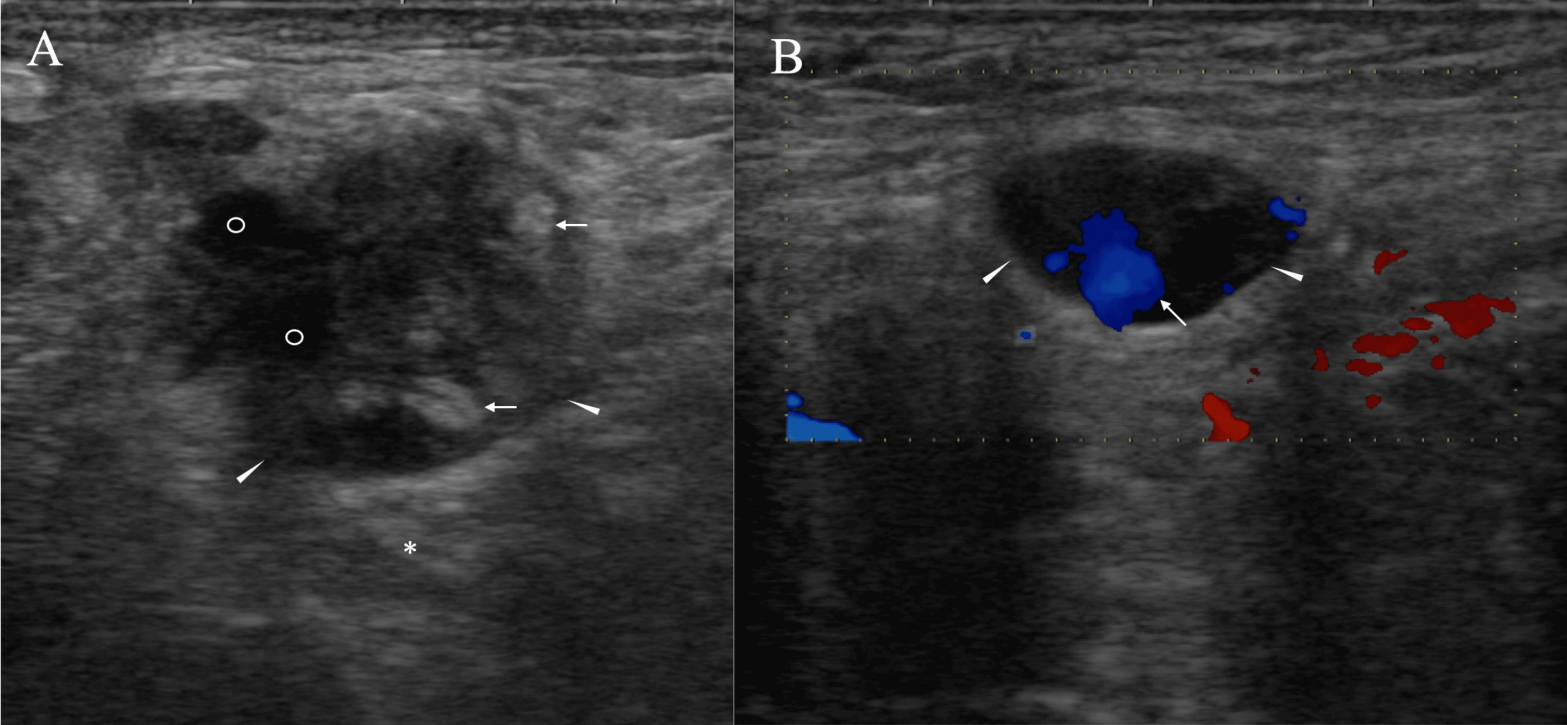
Ultrasound findings A) Ultrasound shows that one mass (arrowheads) has predominant internal high echoes, small very low-echo areas (open circles), and enhanced posterior echoes (asterisk). Very strong high-echo areas (arrows) appear to correspond to the focal adipocyte cluster areas. B) Ultrasound shows that another mass (arrowheads) has blood flow (arrow) into the mass.

An 86-year-old man with a Brinkmann index of 600 was referred to our hospital for the evaluation of hematuria three years earlier. Imaging and pathological evaluation led to the diagnosis of bladder cancer with invasion to the surrounding tissue, and the patient underwent radical cystectomy and urinary diversion without adjuvant chemotherapy, due to his advanced age. During postoperative follow-up, the patient complained of pain around the left shoulder. Ultrasound showed multiple round masses, 33 mm in maximal size, extending from the left neck to the left axilla, some of which had fused to form large masses. Ultrasound further clarified that these masses had mixed high and low internal echoes, enhanced posterior echoes, and blood flow into the masses (Figure 1).

**Figure 2:**
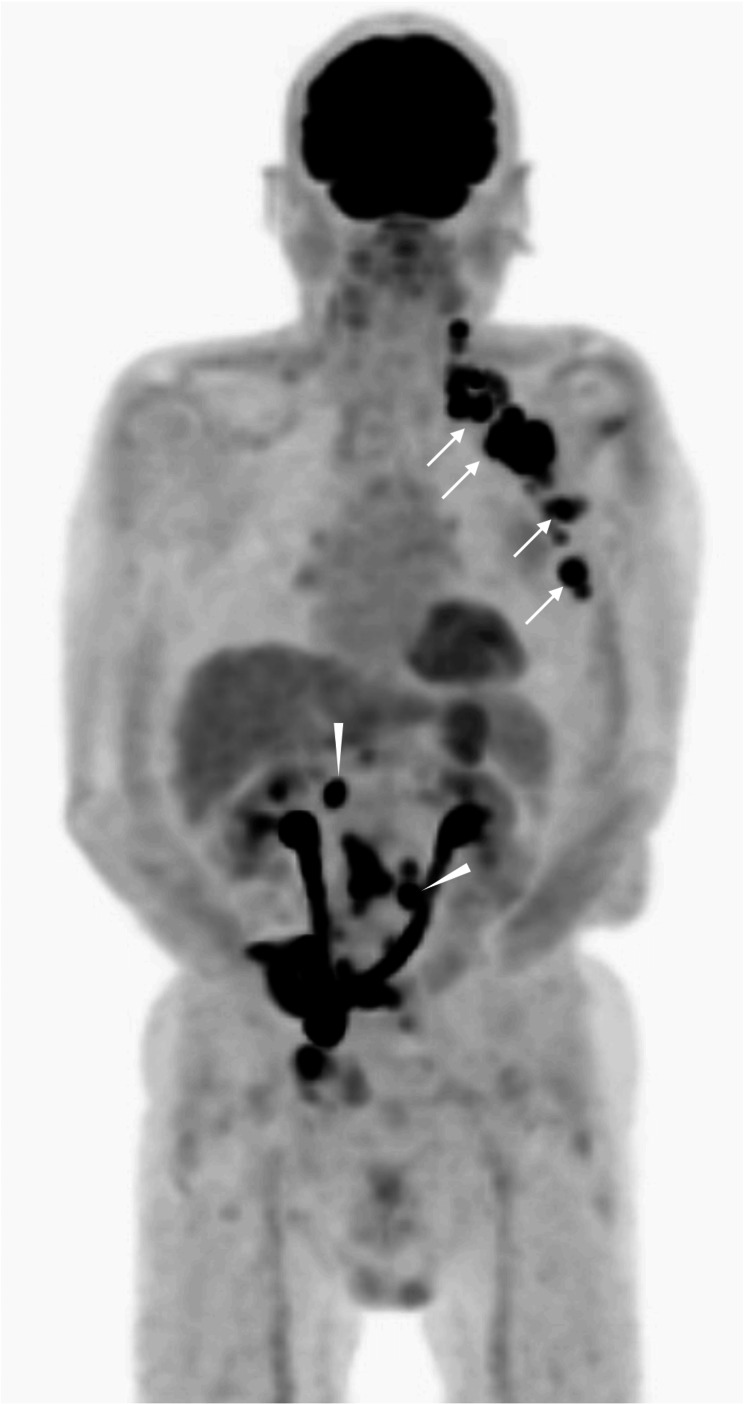
Positron emission tomography (PET) findings PET shows very strong uptake in the left (peri)axillary foci (arrows) and intra-abdominal lesions (arrowheads), and no uptake in the regions around the right axilla.

Positron emission tomography/computed tomography (PET/CT) showed avid fluorodeoxyglucose (FDG) uptake - i.e., a maximal standard uptake value (SUVmax) of 19 - in both the presumed (peri)axillary lymph nodes and the intra-abdominal foci (Figure 2).

**Figure 3:**
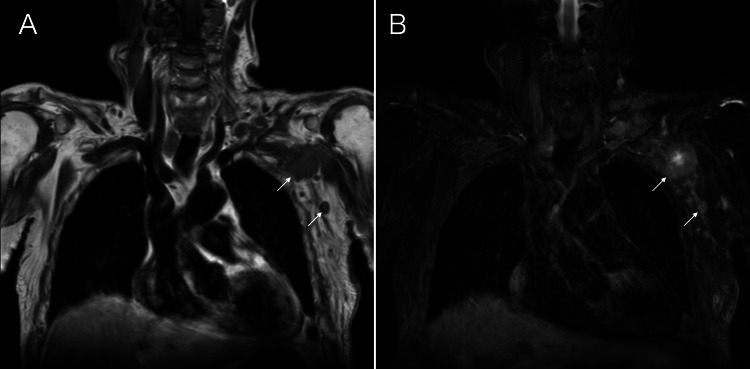
Magnetic resonance imaging (MRI) findings MRI of the axillary masses shows low signals on T1-weighted images (A, arrows) and predominantly very weak high signals on fat-suppressed T2-weighted images (B, arrows).

Magnetic resonance imaging of the (peri)axillary nodes showed low signals on T1-weighted images, and predominantly very weak high signals on fat-suppressed T2-weighted images (Figure 3).

**Figure 4:**
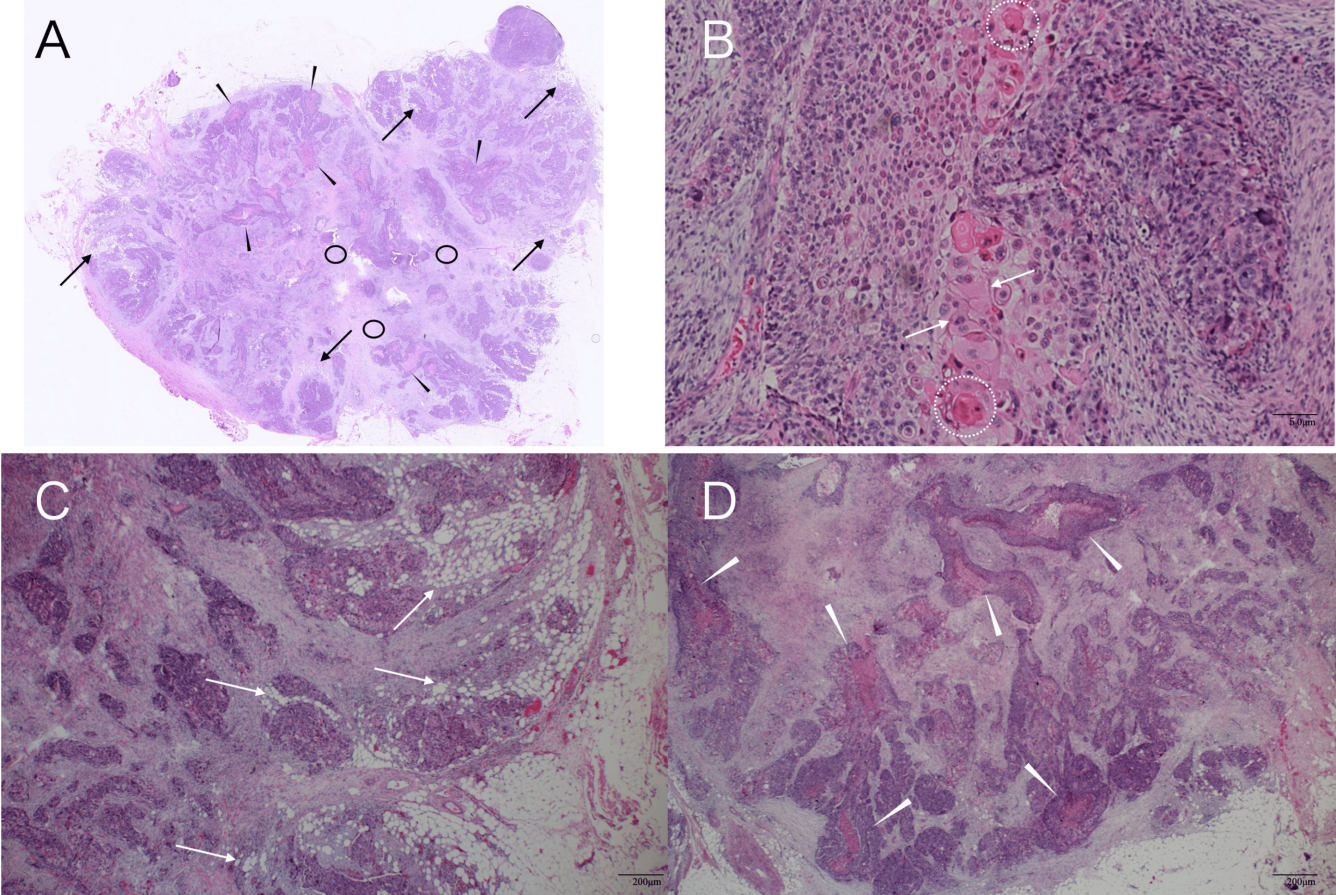
Pathological findings A) Low magnified view showed adipocyte clusters in small areas (arrows), many tubular structures (arrowheads), and hypo-cellular areas (circles). B) Magnified view (H&E, ×200) showed cancer pearls (dotted circles) and intercellular bridges (arrows). C) Magnified view (H&E, ×40) showed adipocyte clusters (arrows) near the tumor borders. D) Magnified view (H&E, ×40) showed many tubular structures (arrows).

Blood tests showed an elevated squamous cell carcinoma antigen (SCC) level of 9.5 ng/mL (reference range: 0-1.5 ng/mL) and a mildly elevated soluble interleukin-2 receptor (sIL-2R) level of 851 U/mL (reference range: 122-496 U/mL) (Table 1). Ultrasound findings strongly suggested the recurrence of bladder cancer in the (peri)axillary lymph nodes, but could not rule out possible malignant lymphoma due to the elevated sIL-2R level, prompting us to biopsy at least one axillary lymph node. We, therefore, performed an excisional biopsy not of the fused, very large lymph node, but of the enlarged yet easily resectable lymph node in the axilla. Frozen section analysis showed no lymphoma cells, but atypical cells proliferating in a solid fashion with keratinization and intercellular bridges (Figure 4). 

**Table 1:** Laboratory tests AST: aspartate aminotransferase; ALT: alanine aminotransferase; LDH: lactate dehydrogenase; ALP: alkaline phosphatase; γ-GTP: γ-glutamyl transpeptidase; Ch-E: cholinesterase; CK: creatine kinase; CRP: C-reactive protein; WBC: white blood cell count; RBC: red blood cell count; Hb: hemoglobin; Ht: hematocrit; CEA: carcinoembryonic antigen; SCC: squamous cell carcinoma antigen; sIL-2R: soluble interleukin-2 receptor

Test	Reference range	Result
Total bilirubin	0.4-1.5 mg/dL	0.5
AST	13-30 U/L	18
ALT	7-23 U/L	9
LDH	124-222 U/L	210
ALP	38-113 U/L	74
γ-GTP	9-32 U/L	21
Ch-E	201-421 U/L	166
CK	41-153 U/L	65
Amylase	33-132 U/L	103
Total protein	6.6-8.1 g/dL	7.2
Albumin	4.1-5.1 g/dL	3.4
CRP	0-0.14 mg/dL	0.3
WBC	33-86 × 10^2^/μL	83
RBC	386-492 × 10^4^/μL	396
Hb	11.6-14.8 g/dL	11.8
Ht	35.1-44.4%	37.4
Platelet	15.8-34.8 × 10^4^/μL	28.3
CEA	0-5.0 ng/mL	2.2
SCC	0-1.5 ng/mL	9.5
sIL-2R	122-496 U/mL	851

**Figure 5:**
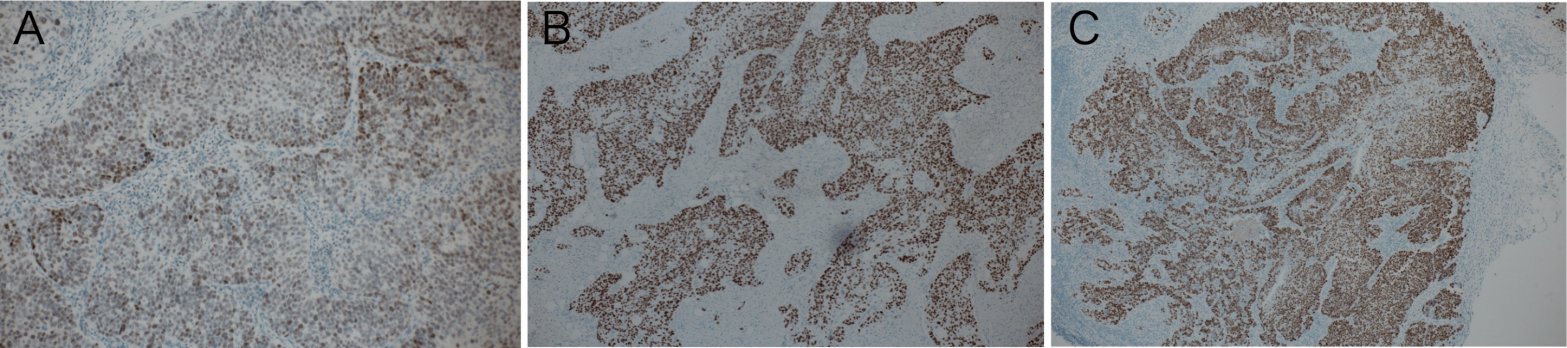
Immunostaining findings Immunostaining shows that the tumor cells were positive for squamous cell carcinoma markers, such as GATA3 (A), p40 (B), and CK5/6 (C).

Immunostaining showed GATA3, p40, and CK5/6 positivity (Figure 5), leading to the diagnosis of SCC metastasis to the lymph nodes.

Due both to his old age and the patient’s preference, the patient did not receive chemotherapy and only underwent radiotherapy to the supra-clavicular, axillary, and intra-abdominal foci for disease control - unfortunately resulting in the discontinuation of radiotherapy due to severe side effects, including grade 2 diarrhea. The patient, therefore, has been receiving best supportive care for three months after the excisional biopsy.

## Discussion

Various tumor markers can contribute to the diagnosis of malignant disorders, including malignant lymphoma. Both sIL-2R and SCC were elevated in this case and are well known to be useful for the diagnosis of malignant lymphoma and bladder cancer, respectively. The sIL-2R elevation, however, was relatively mild at 851 U/mL, which can be observed not only in malignant lymphoma patients but also in patients with various pathological conditions, such as allergic reactions and sarcoidosis caused by T cell activation [9]. In contrast, the elevated SCC level of 9.5 ng/mL was very high and strongly suggested bladder cancer recurrence in this case [10].

Enlarged lymph nodes in the (peri)axillary region had a relatively high proportion of internal high-echo areas. The high SUVmax value of 19 led us to assume that the masses have very aggressive characteristics. Expansive mass growth observed in malignant lymphomas generally implies cellular uniformity due to their high proliferative ability [11]. This fact suggests the similarity of acoustic impedance among lymphoma cells and minimal ultrasound wave backscattering, which generally makes the internal echoes of malignant lymphomas very low [12].

It is well known that the presence of adipocytes in the masses generates much stronger internal high echoes than pathological structure-induced high echoes, such as microvoid- or mesh structure-containing components. In addition, we have already found that malignant lymphomas can sometimes have high internal echoes due to the presence of adipocytes [13], which were also found sparsely and focally in very small areas of the resected mass in this case. Major parts of the internal high echoes, however, were much less intense than the presumed adipocyte-induced high echoes in this case. Furthermore, if the presence of fat cells within the tumor had caused the weak high signals of the tumor on fat-suppressed T2-weighted images, the tumor should have shown high signals on T1-weighted images. We, therefore, can conclude that the (peri)axillary masses were not caused by malignant lymphoma, but by bladder cancer metastasis to the lymph nodes, even based on image evaluation. In short, bladder cancer cells were transported through the thoracic duct to the left venous angle and spread to the lymph nodes around the venous angle in this case.

PET generally shows strong accumulation of FDG in both the brain, which consumes more glucose, and the urinary tract, where FDG is metabolized, even in healthy persons. Furthermore, inflammation also shows FDG accumulation but never reaches an SUVmax level of 19. Image evaluation mentioned above, therefore, leads us to the judgment that the internal high-echo areas cannot be formed by malignant lymphomas. In fact, pathological study clarified that the excised lymph node had many tubular structures capable of generating ultrasound wave backscattering [7,8], and scattered minimal adipocyte clusters only in very limited areas. Diagnostic physicians, therefore, should not immediately perform an excisional biopsy, but instead perform a core needle biopsy of the target lesion when observing image findings like those in this case to avoid unnecessary harm to the patients. In addition, it is very important for diagnostic physicians to be familiar with the basic mechanisms of ultrasound image formation to accurately predict the pathological findings of the internal high echoes [13].

## Conclusions

High SUVmax values can strongly suggest tumor aggressiveness and generally lead to very low internal-echo formation in malignant lymphomas. Therefore, tumors with high SUVmax values and high internal echoes are exceptional in malignant lymphomas. Diagnostic physicians should not perform an immediate excisional biopsy, but rather perform a core needle biopsy of the target lesion when these image findings are present, to avoid unnecessary harm to the patients.
